# Biodistribution, shedding, and transmissibility of vusolimogene oderparepvec (RP1)

**DOI:** 10.3389/fonc.2026.1798502

**Published:** 2026-07-28

**Authors:** Trisha M. Wise-Draper, Caroline Robert, Michael K. Wong, Mark R. Middleton, Joseph J. Sacco, Gino K. In, Eva Muñoz Couselo, Dirk Schadendorf, Georgia M. Beasley, Jiaxin Niu, Bartosz Chmielowski, Mohammed M. Milhem, Tawnya Lynn Bowles, Katy K. Tsai, Adel Samson, Kevin J. Harrington, Céleste Lebbé, Caroline Gaudy-Marqueste, Junhong Zhu, Bhavna Paratala, Jeannie W. Hou, Kostas Xynos, Aaron Clack, Robert S. Coffin, Praveen K. Bommareddy

**Affiliations:** 1 University of Cincinnati Cancer Center, University of Cincinnati, Cincinnati, OH, United States; 2 Gustave Roussy and Paris-Saclay University, Villejuif, France; 3 Roswell Park Comprehensive Cancer Center, Buffalo, NY, United States; 4 Churchill Hospital, University of Oxford, Oxford, United Kingdom; 5 The Clatterbridge Cancer Centre, Wirral, United Kingdom; 6 University of Liverpool, Liverpool, United Kingdom; 7 University of Southern California Norris Comprehensive Cancer Center, Los Angeles, CA, United States; 8 Vall d’Hebron Institute of Oncology (VHIO) and Vall d’Hebron Hospital Medical Oncology Department, Barcelona, Spain; 9 Department of Dermatology, West German Cancer Center, University Hospital Essen, Essen & National Center for Tumor Diseases (NCT-West), University Alliance Ruhr, Research Alliance Ruhr, Research Center One Health, University Duisburg-Essen, Essen, Germany; 10 Duke Cancer Institute, Duke University, Durham, NC, United States; 11 Banner MD Anderson Cancer Center, Gilbert, AZ, United States; 12 Jonsson Comprehensive Cancer Center, University of California, Los Angeles, Los Angeles, CA, United States; 13 Holden Comprehensive Cancer Center, University of Iowa, Iowa City, IA, United States; 14 Intermountain Medical Center, Murray, UT, United States; 15 Helen Diller Family Comprehensive Cancer Center, University of California, San Francisco, San Francisco, CA, United States; 16 Leeds Institute of Medical Research at St. James’s, University of Leeds, Leeds, United Kingdom; 17 The Institute of Cancer Research/Royal Marsden NIHR Biomedical Research Centre, London, United Kingdom; 18 Université Paris Cité, AP-HP Dermato-Oncology and CIC, Cancer Institute APHP. Nord–Université Paris Cité, INSERM U976, Saint Louis Hospital, Paris, France; 19 Aix-Marseille Université, APHM, Centre de Recherche en Cancérologie de Marseille (CRCM), INSERM, U1068, CNRS, UMR7258, UM105, Hôpital Timone, CEPCM, Dermatology and Skin Cancer Department, Marseille, France; 20 Replimune, Inc., Woburn, MA, United States

**Keywords:** biodistribution and shedding, RP1, PD-1 failed melanoma, Vusolimogene oderparepvec (RP1), oncolytic immunotherapy, HSV-1, Replimune

## Abstract

**Background:**

Vusolimogene oderparepvec (RP1) is an intratumorally administered, genetically modified herpes simplex virus type-1 derived oncolytic immunotherapy designed to selectively replicate in tumors and stimulate systemic antitumor immunity. We evaluated the biodistribution, shedding, and potential for transmission of RP1 in patients with skin cancers treated in the IGNYTE clinical trial and assessed close-contact exposure across all RP1 clinical studies.

**Methods:**

Patients received intratumoral RP1 in combination with nivolumab, with serial collection of blood, urine, injection-site, dressing, and oral mucosal samples during treatment and follow-up. RP1 DNA was quantified by polymerase chain reaction, and swab samples positive for RP1 DNA were tested for replication-competent RP1. Reports of herpetic infection in patients and close contacts were also systematically collected.

**Results:**

Among 282 treated patients, RP1 DNA was detected most frequently at injection sites, with substantially lower incidence and levels in other sample types. Detection declined rapidly after treatment completion, with no RP1 DNA detected in blood or urine during follow-up. Replication-competent RP1 was detected rarely and only at low titers, only at injection sites. No systemic herpes simplex virus infections occurred in patients, and no herpetic infections were reported among caregivers or close contacts.

**Interpretation:**

RP1 is largely confined to injection sites, shedding of live RP1 is rare and transient. No evidence of transmission of RP1 was observed. These findings support the favorable biosafety profile of RP1 with only a negligible risk of environmental release or secondary transmission during clinical use.

## 1 Introduction

Oncolytic viral immunotherapies are a class of therapeutic agents which selectively replicate in and lyse tumors, while at the same time stimulating a systemic antitumor immune response (1). They have been used successfully as both standalone treatments and in combination with immune checkpoint blockade (2, 3). Vusolimogene oderparepvec (VUSO, RP1) is an oncolytic immunotherapy derived from herpes simplex virus-1 (HSV-1) strain RH018A which has deletions of the ICP34.5- and ICP47-encoding genes to provide tumor selectivity and which expresses both human granulocyte-macrophage colony-stimulating factor (GM-CSF) and the gibbon ape leukemia virus (GALV-GP R-) fusogenic glycoprotein (4) to promote immunogenic cell death and immune infiltration. In the IGNYTE Phase 2 clinical trial in anti-PD-1 failed melanoma, a 33% response rate with strong durability (median, 24.8 months) was shown in 140 patients treated with RP1 in combination with nivolumab. Similar tumor reductions (>30%) were observed in both injected and non-injected lesions, including visceral lesions (3).

As RP1 is derived from HSV-1, there is a potential risk of transfer to healthcare providers and other patient contacts during treatment and after administration (5). Although the deletions in RP1 render it unable to replicate productively in non-tumor tissue, there is a theoretical possibility of RP1 transmission following patient treatment. Notably, the HSV-1 thymidine kinase gene is intact in RP1 and thus retains sensitivity to acyclovir (6), which could be utilized in the unlikely event of a clinical manifestation of RP1 infection being seen. An objective of the current study was to provide a systematic evaluation of RP1 biodistribution and shedding, assessing and quantifying both RP1 DNA and live RP1 in swab samples from injection sites, injection site dressings and mucosa, and RP1 DNA only in blood and urine in the IGNYTE study. Additionally, the study also assessed the potential for transmission of RP1 to patient contacts during and after therapy.

## 2 Materials and methods

### 2.1 Study design

**Figure 1:**
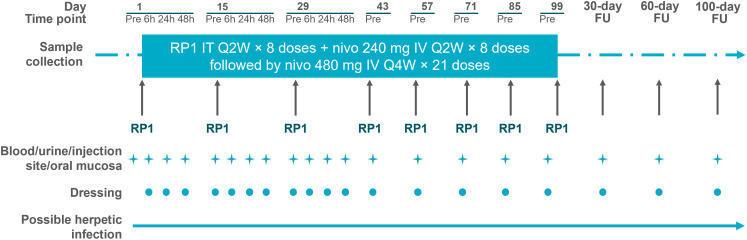
The IGNYTE study design and sample collection schedule. FU, follow-up; h, hours; IT, intratumoral; IV, intravenous; nivo, nivolumab; Pre, pre-dose; Q2W, every 2 weeks; Q4W, every 4 weeks. RP1 is given at starting dose of 10e^6^PFU/mL flowed by 10e^7^ PFU/mL from doses 2-8.

In the phase 2 portion of the IGNYTE study, patients received an initial dose of RP1 at 1×10^6^ PFU/mL, followed by subsequent doses of 1×10^7^ PFU/mL every 2 weeks across multiple cohorts with different tumor types (including, melanoma and non-melanoma skin cancers). Patients were required to have at least one measurable tumor lesion assessable by RECIST and a cumulative injectable tumor size by unidimensional measurement of 1 cm for RP1 injection, with a maximum allowable injection volume of 10 mL per visit. Multiple tumors could be injected within the 10 mL volume limit per treatment session, including by imaging guidance. Nivolumab treatment commenced from the second RP1 dose and was initially administered at 240 mg every two weeks for four months; this was followed by nivolumab 480 mg every four weeks, which was continued until confirmed disease progression, intolerable toxicity, or up to two years, whichever occurred sooner. During RP1 treatment, blood, urine, and swab samples (from saliva/oral mucosa, injection sites, and injection-site dressings) were collected for RP1 DNA detection. For swab samples, any samples testing positive for RP1 DNA by qPCR were tested for the presence of live RP1 using the TCID50 assay. The sample collection schedule is summarized in **Figure 1**.

### 2.2 Patients

The patients included in this analysis were those from the skin cancer cohorts in the IGNYTE clinical trial (n=282), including an initial melanoma cohort (n=30), the anti-PD-1 failed registration intended cutaneous melanoma cohort (n=141), the anti-PD-1 naïve non-melanoma skin cancer (NMSC) (n=32), and the anti-PD-1 failed NMSC cohort (n=79).

### 2.3 Sample collection

The biodistribution and shedding of RP1 were assessed using a quantitative polymerase chain reaction (qPCR) assay, following current guidelines (7, -ICH considerations—general principles to address virus and vector shedding, 8, -Design and Analysis of Shedding Studies for Virus or Bacteria-Based Gene Therapy and Oncolytic Products, 9- Design and Analysis of Shedding Studies for Virus or Bacteria-Based Gene Therapy and Oncolytic Products, Guidance for Industry). The timepoints and sample types collected were based on FDA Guidance, 2015, and ICH guideline, 2009 (7–9). Samples were collected prior to dosing during the first to eighth dose of RP1, and at 30, 60, after the final RP1 injection and 100 days post final nivolumab dosing. Blood, urine, and swabs from the injection sites, dressings, and oral mucosa were collected pre-treatment, during treatment, and post-treatment. Additional early timepoint samples (6 ± 2, 21 ± 3, and 48 ± 6 hours after the first three RP1 injections) were collected in a subset of approximately 6 patients per Phase 2 tumor cohort, with four cohorts representing skin cancer indications. Variability in sample collection resulted in slightly different numbers at each timepoint.

This more frequent early timepoint sample collection was performed for all Phase 1 dose-escalation and expansion patients (not reported here), and provided the initial safety and shedding characterization. Following Phase 1, a reduced sampling strategy which was implemented in Phase 2 to reduce the workload for trial sites and to reduce the intensity of procedures for patients.

Additionally, to evaluate the potential infectivity of RP1, swab samples were collected from any suspicious areas that appeared clinically to be of potentially herpetic origin. Blood samples were also collected if a patient experienced unexplained febrile illness of Grade 3 or higher. All swab samples that tested positive for RP1 DNA were further tested for the presence of live RP1 by tissue culture infectious dose 50 (TCID50) assay (10). This test was only validated for swab samples and not for urine or blood as presence of various contaminates could interfere with the assay. All samples were shipped on dry ice to Eurofins Viracor BioPharma (Lenexa, KS) and stored at -80 °C until testing.

### 2.4 Close contact collection

Across all RP1 studies, the IGNYTE study, a randomized, controlled, open-label, phase 2 study of cemiplimab ± RP1 in patients with advanced cutaneous squamous cell carcinoma (CERPASS) (11), and RP1 monotherapy in solid organ and hematopoietic cell transplant recipients with skin cancers (ARTACUS) (12), patients were provided with a questionnaire about symptoms of HSV-1 infection to close contacts or caregivers during each study visit.

### 2.5 Quantitative polymerase chain reaction

DNA was extracted from blood, urine, and swab samples using standard methods (QIAcube from Qiagen, Netherlands, for blood and Nuclisens easyMAG from BioMérieux, France, for urine and swab samples), and any RP1 DNA was amplified via a TaqMan qPCR assay (Thermo Fisher Scientific, Waltham, MA) with primer/probe sequences that are specific for the cytomegalovirus (CMV)-Rous sarcoma virus (RSV) promoter junction present in two copies in the RP1 genome (4). This qPCR assay does not cross-react with wild-type HSV-1 or CMV. A linearized plasmid control was serially diluted to generate a standard curve, which was used to quantify RP1 DNA in the test samples. Detection was reported as copies/mL for blood and urine samples, and copies/swab for swab samples. Negative thresholds were set at ≤ 41 copies/mL for blood, ≤ 53 copies/mL for urine, and ≤ 110 copies/swab based on analytical validation. Samples below quantification limits (BQL) were assigned a nominal value of zero, with BQL defined as ≤ 51 copies/mL for blood, ≤ 100 copies/mL for urine, and ≤ 150 copies/swab. Detection less than the limit of quantitation (LOQ) was considered positive but not quantifiable.

### 2.6 Tissue culture infections dose (TCID50)

The TCID50 assay was used to assess the concentration of RP1 (PFU/mL) at which 50% of Vero cells (African green monkey kidney cells) exhibit a cytopathic effect which is then translated to plaque forming units (PFU). Swabs that were BQL in the qPCR assay were generally not tested in the TCID50 assay, except for follow-up samples or those with indeterminate results. Vero cells were seeded into 96-well plates, and a dilution series of the original sample buffer (diluted in phosphate-buffered saline) was applied to infect the cells. A dilution series of positive control samples with known RP1 titers was also included. After incubation, the number of wells exhibiting cytopathic effects was recorded, and these results were used to calculate the live RP1 titer. The TCID50 assay was optimized and validated at Eurofins Viracor Biopharma.

## 3 Results

### 3.1 Baseline characteristics

Baseline characteristics are summarized in **Table 1**. Out of 282 patients included in the study, the majority were White (80.9%), with smaller proportions identifying as Black or African American (1.8%), Asian (0.7%), and American Indian or Alaska Native (0.4%). Race was not reported for 16.3% of patients. The cohort was predominantly male (69.5%). The median age was 66.0 years (range 21–97), with 47.9% of patients aged 18–64 years, 30.1% aged 65–74 years, and 22.0% aged 75 years or older. ECOG performance status was available for all patients, with 59.9% classified as ECOG 0 and 39.4% ECOG 1 and 0.7% ECOG 2.

**Table 1:** Patient demographics and baseline characteristics.

Baseline character	N(%)
Age, years	
N	282
Mean (StD)	64.3 (13.66)
Median	66.0
Min – Max	21.0-97.0
Age group, years, n(%)	
18-64	135 (47.9)
65-74	85 (30.1)
>=75	62 (22.0)
Sex, n (%)	
Male	196 (69.5)
Female	86 (30.5)
Race, n (%)	
American Indian Or Alaska Native	1 (0.4)
Asian	2 (0.7)
Black Or African American	5 (1.8)
White	228 (80.9)
Not Reported	46 (16.3)
Region, n (%)	
United States	171 (60.6)
Non-United States	111 (39.4)
ECOG Performance, n (%)	
0	169 (59.9)
1	111 (39.4)
2	2 (0.7)

ECOG PS, Eastern Cooperative Oncology Group performance status. Non-United States include United Kingdom, France, Spain, and Germany.

### 3.2 Baseline and post-treatment HSV-1 IgG serostatus

At baseline, the HSV-1 IgG serostatus of patients enrolled in the IGNYTE skin cancer cohorts was assessed. Among the total 282 patients, consistent with expectations for the general population (13), 203 of 282 patients (72.0%) were found to be seropositive for HSV-1 IgG antibodies, while 73 patients (25.9%) tested seronegative. The serostatus of 6 patients (2.1%) was indeterminate or unknown at the time of baseline assessment (**Table 2**). Most seronegative patients seroconverted by Cycle 3 Day 29 after treatment with RP1. The serostatus of patients from Cycle 1 to Cycle 4 is shown below (**Table 2**).

**Table 2:** HSV-1 serostatus at baseline and post treatment with RP1.

Visit	HSV-1 seropositive n (%)	HSV-1 seronegative n (%)	HSV-1 unknown n (%)
Cycle 1 Day 1	203 (72.0)	73 (25.9)	6 (2.1)
Cycle 2 Day 15	216 (76.6)	31 (11.0)	35 (12.4)
Cycle 3 Day 29	235 (83.3)	9 (3.2)	38 (13.5)
Cycle 4 Day 43	222 (78.7)	5 (1.8)	55 (19.5)

n, number of patients with samples collected. Percentage is calculated based on the total number of subjects for the Cohort. For post baseline values a missing numerical value with a qualitative result as positive is considered positive, if the visit prior has result > 1.

### 3.3 RP1 DNA detection in blood

**Figure 2:**
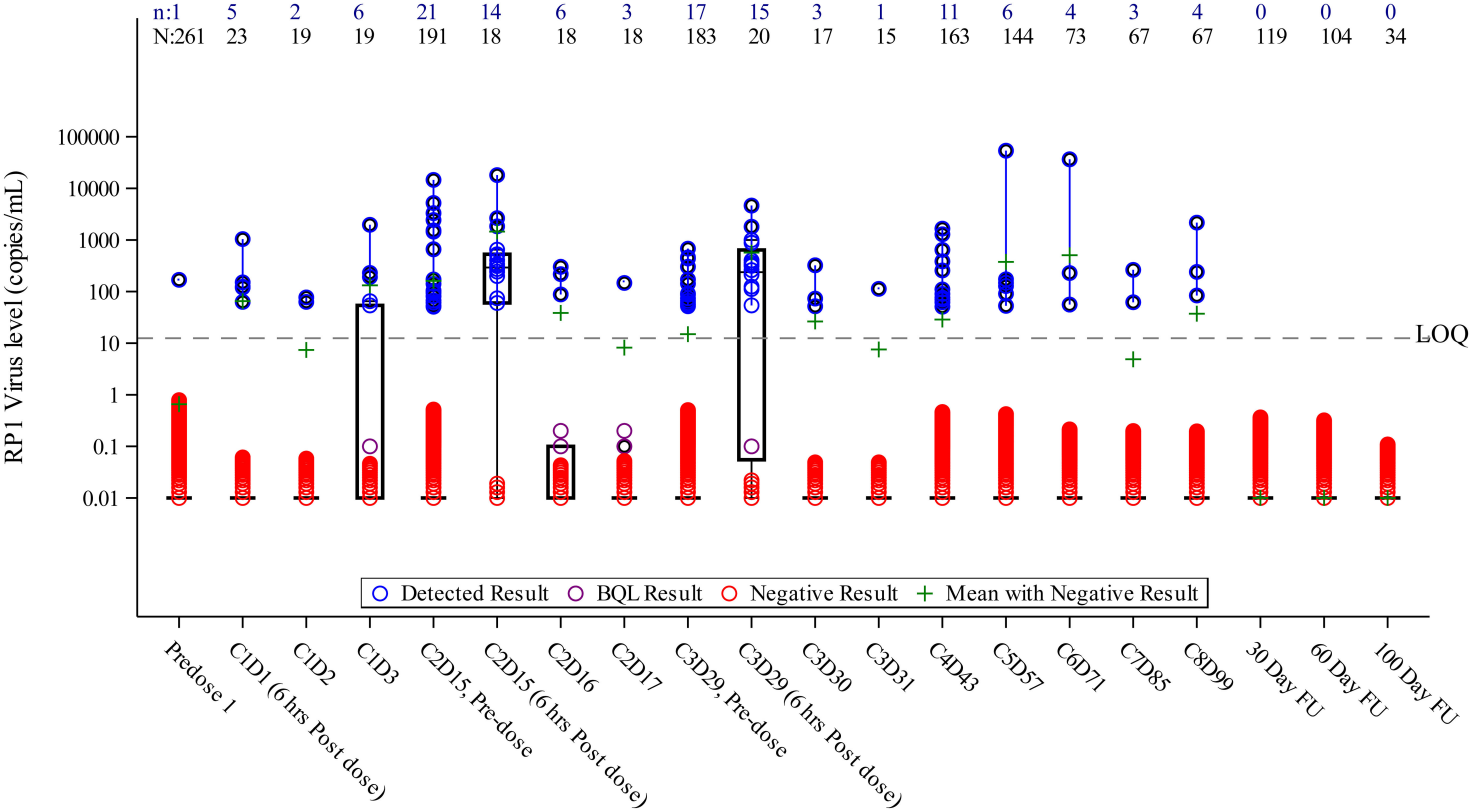
RP1 DNA detection in blood. RP1 DNA detection in blood samples from all skin cancer cohorts across various study time points. “n” represents the number of patients with viral DNA levels equal to or above the limit of quantification (LOQ), while “N” indicates the total number of patients assessed at each time point. The study includes measurements taken at pre-dose, multiple post-dose time points, and follow-up visits, including Cycle 1 Day 1 (C1D1), Cycle 2 Day 15 (C2D15), Cycle 3 Day 29 (C3D29), Cycle 4 Day 43 (C4D43), Cycle 5 Day 57 (C5D57), Cycle 6 Day 71 (C6D71), Cycle 8 Day 99 (C8D99), 30 Day follow-Up (FU), 60 Day FU and 100 Day FU. Individual data points include detected results (blue circles), below quantification limit (BQL) results (purple circles), and negative results (red circles). Green plus signs represent the mean virus levels.

RP1 DNA was detected in the blood of 53 of 274 (19.3%) patients and in 122 of 1573 (7.8%) samples during treatment. The highest levels of RP1 DNA were observed within 6 hours of injection, with levels decreasing thereafter (**Table 3**; **Figure 2**; **Supplementary Figure 1**). One sample tested positive for RP1 DNA at pre-dose Cycle 1 Day 1, likely due to contamination, as no RP1 was administered prior to that visit. Only a small subset (5.5%) of patients continued to have RP1 DNA detected in the blood until the next RP1 dose (15 days later). RP1 DNA was rarely detected in blood (~5%) beyond the fifth dose of RP1.

**Table 3:** Patient and sample incidence of RP1 DNA detection in blood.

	Patient incidence					Sample incidence			
	Baseline HSV-1 seronegative N=72 n1/n2 (%)	Baseline HSV-1 seropositive N=200 n1/n2 (%)	Baseline HSV-1 unknown N=6 n1/n2 (%)	Overall N=278 n1/n2 (%)		Baseline HSV-1 seronegative n3/n4 (%)	Baseline HSV-1 seropositive n3/n4 (%)	Baseline HSV-1 unknown n3/n4 (%)	Overall n3/n4 (%)
Blood	25/70 (35.7)	27/198 (13.6)	1/6 (16.7)	53/274 (19.3)		76/444 (17.1)	45/1091 (4.1)	1/38 (2.6)	122/1573 (7.8)

N, Number of patients in the analysis set; n1, number of patients with positive qPCR testing result; n2, number of patients with samples collected; n3, number of samples with positive qPCR testing result; n4, number of samples collected.

The incidence of RP1 DNA detection from blood samples was higher in baseline seronegative patients, with 25 of 70 (35.7%) patients and 76 of 444 (17.1%) samples testing positive, compared to 27 of 198 (13.6%) patients and 45 of 1091 (4.1%) samples in baseline seropositive patients (**Table 3**; **Supplementary Figure 1**). Circulating RP1 in blood was rapidly cleared by the immune response in seropositive patients, whereas at early timepoints (before seropositivity), it was less quickly cleared and therefore detectable levels were maintained between injections (**Supplementary Figure 2**). RP1 DNA detection in blood samples declined over time post treatment, and RP1 DNA was no longer detectable by the 30, 60 and 100 days at follow up visits, demonstrating complete clearance of RP1 DNA from the systemic circulation whether or not a patient was HSV-1 seropositive at baseline (**Table 3**; **Figure 2**).

### 3.4 RP1 DNA detection in urine

**Figure 3:**
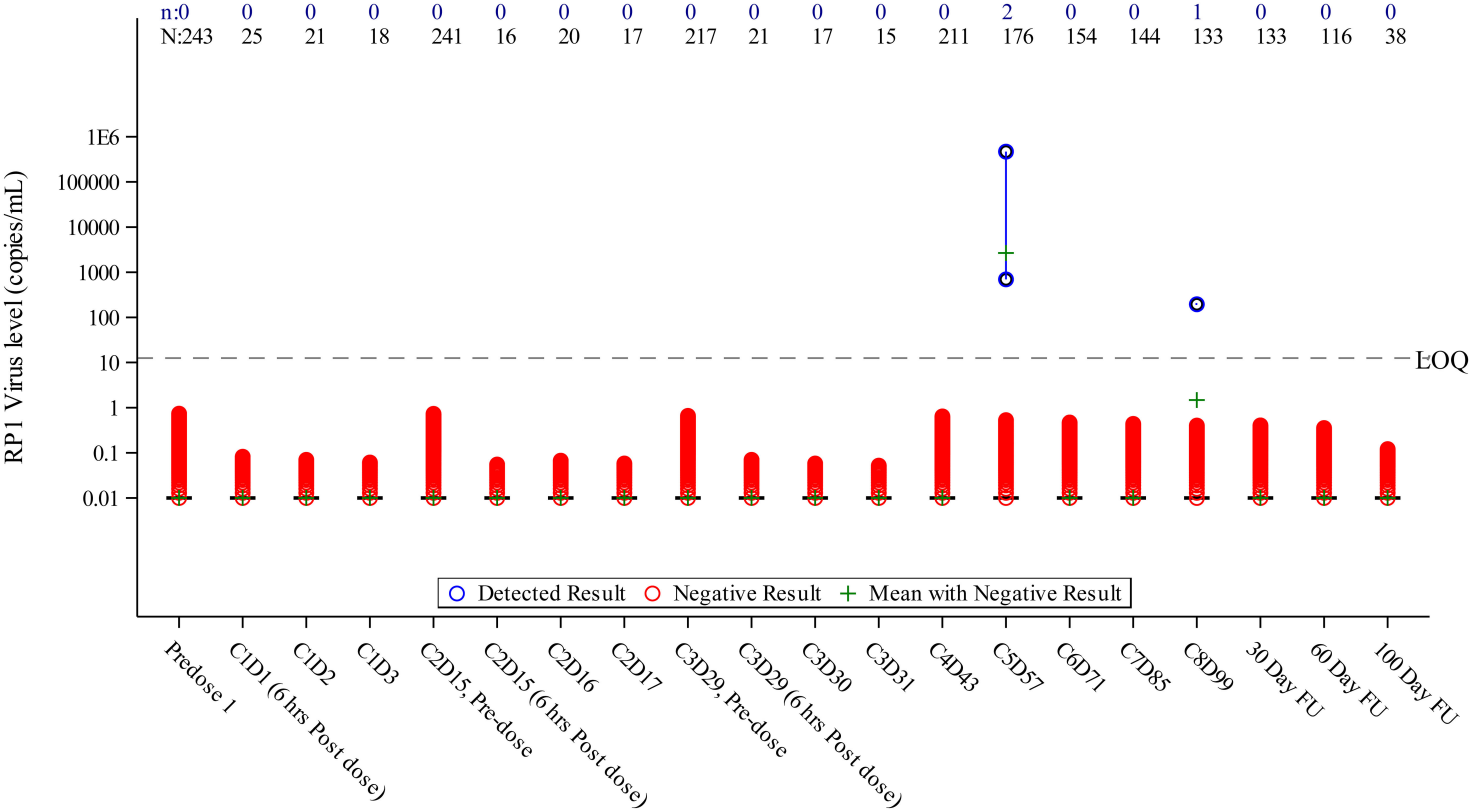
RP1 DNA detection in urine. RP1 DNA detection in urine samples from all skin cancer cohorts across various study time points. “n” represents the number of patients with viral DNA levels equal to or above the limit of quantification (LOQ), while “N” indicates the total number of patients assessed at each time point. The study includes measurements taken at pre-dose, multiple post-dose time points, and follow-up visits, including Cycle 1 Day 1 (C1D1), Cycle 2 Day 15 (C2D15), Cycle 3 Day 29 (C3D29), Cycle 4 Day 43 (C4D43), Cycle 5 Day 57 (C5D57), Cycle 6 Day 71 (C6D71), Cycle 8 Day 99 (C8D99), 30 Day follow-Up (FU), 60 Day FU and 100 Day FU. Individual data points include detected results (blue circles), below quantification limit (BQL) results (purple circles), and negative results (red circles). Green plus signs represent the mean virus levels.

The detection of RP1 DNA in urine was minimal, with only 0.7% (2/273) of patients and 0.2% (3/1976) of samples testing positive (**Table 4**; **Figure 3**; **Supplementary Figure 1**). No RP1 DNA was detected in urine samples between the first and fourth dose. The few samples that tested positive were collected two weeks after the fourth and seventh doses and then tested negative on the next visit 15 days later.

**Table 4:** Patient and sample incidence of RP1 DNA detection in urine.

	Patient incidence					Sample incidence			
	Baseline HSV-1 seronegative N=72 n1/n2 (%)	Baseline HSV-1 seropositive N=200 n1/n2 (%)	Baseline HSV-1 unknown N=6 n1/n2 (%)	Overall N=278 n1/n2 (%)		Baseline HSV-1 seronegative n3/n4 (%)	Baseline HSV-1 seropositive n3/n4 (%)	Baseline HSV-1 unknown n3/n4 (%)	Overall n3/n4 (%)
Urine	1/71 (1.4)	1/196 (0.5)	0/6 (0.0)	2/273 (0.7)		1/567 (0.2)	2/1374 (0.1)	0/35 (0.0)	3/1976 (0.2)

N, Number of patients in the analysis set; n1, number of patients with positive qPCR testing result; n2, number of patients with samples collected; n3, number of samples with positive qPCR testing result; n4, number of samples collected.

RP1 DNA was detected in 1 of 71 (1.4%) patients and 1 of 567 (0.2%) samples in baseline seronegative patients, while 1 of 196 (0.5%) patients and 2 of 1374 (0.1%) samples tested positive in baseline seropositive patients (**Table 4**; **Supplementary Figure 1**). Of the two patients of whom RP1 DNA was detected in urine, one was HSV-1 seronegative, and one was seropositive at baseline, precluding any meaningful interpretation of differences by serostatus. No RP1 DNA was detected in urine samples collected at the 30-, 60-, and 100-day follow-up visits, confirming clearance (**Table 4**; **Figure 3**).

### 3.5 RP1 detection at the injection site

**Figure 4:**
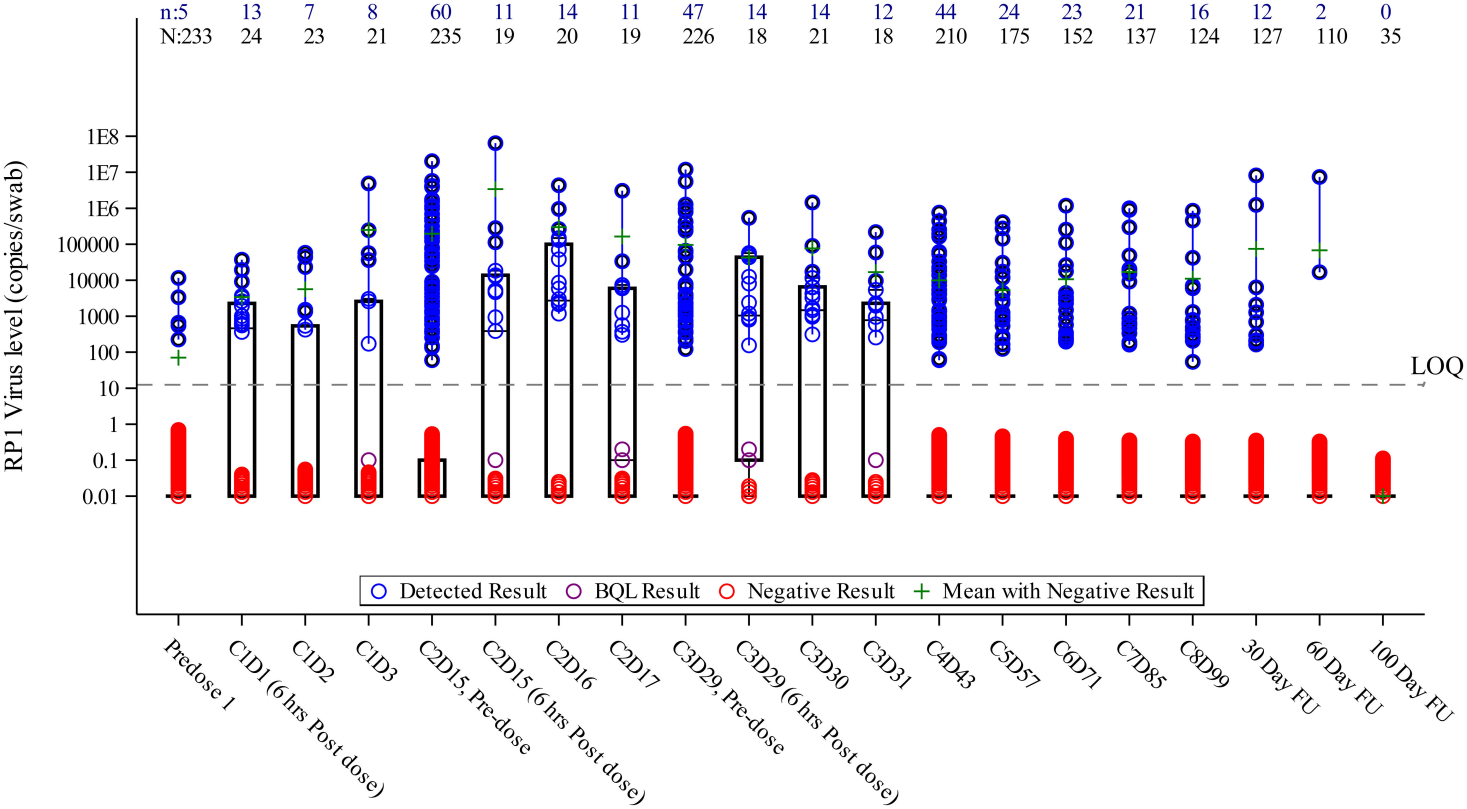
RP1 DNA detection at injection-sites. RP1 DNA detection in injection-site samples from all skin cancer cohorts across various study time points. “n” represents the number of patients with viral DNA levels equal to or above the limit of quantification (LOQ), while “N” indicates the total number of patients assessed at each time point. The study includes measurements taken at pre-dose, multiple post-dose time points, and follow-up visits, including Cycle 1 1 (C1D1), Cycle 2 Day 15 (C2D15), Cycle 3 Day 29 (C3D29), Cycle 4 Day 43 (C4D43), Cycle 5 Day 57 (C5D57), Cycle 6 Day 71 (C6D71), Cycle 8 Day 99 (C8D99), 30 Day follow-Up (FU), 60 Day FU and 100 Day FU. Individual data points include detected results (blue circles), below quantification limit (BQL) results (purple circles), and negative results (red circles). Green plus signs represent the mean virus levels.

RP1 DNA was detected on the surface of injected lesions in 42.1% (112/266) of patients and 18.4% (358/1947) of tested samples, primarily during the treatment course (doses 1 to 8) (**Table 5**; **Figure 4**; **Supplementary Figure 1**). In approximately 35% of patients, RP1 DNA remained detectable 15 days after the previous injection, consistent with replication of RP1 in tumors. No significant differences were observed between HSV-1 seronegative patients (48.5%, 33/68) and samples (22.9%, 122/532) compared to seropositive patients (40.1%, 77/192) and samples (16.8%, 232/1378) (**Table 5**; **Supplementary Figure 1**).

**Table 5:** Patient and sample incidence of RP1 DNA detection at the Injection-site.

	Patient incidence					Sample incidence			
	Baseline HSV-1 seronegative N=72 n1/n2 (%)	Baseline HSV-1 seropositive N=200 n1/n2 (%)	Baseline HSV-1 unknown N=6 n1/n2 (%)	Overall N=278 n1/n2 (%)		Baseline HSV-1 seronegative n3/n4 (%)	Baseline HSV-1 seropositive n3/n4 (%)	Baseline HSV-1 unknown n3/n4 (%)	Overall n3/n4 (%)
Injection Site	33/68 (48.5)	77/192 (40.1)	2/6 (33.3)	112/266 (42.1)		122/532 (22.9)	232/1378 (16.8)	4/37 (10.8)	358/1947 (18.4)

N, Number of patients in the analysis set; n1, number of patients with positive qPCR testing result; n2, number of patients with samples collected; n3, number of samples with positive qPCR testing result; n4, number of samples collected.

A total of 314 RP1 DNA-positive samples were assessed for live RP1, of which only 4 (1.3%) collected from three patients were positive by the TCID50 assay (**Table 6**).

**Table 6:** TCID50 positive samples from injection site.

Patient ID	Visit	Timepoint	qPCR (copies/swab)	TCID50 PFU/mL	Dressing qPCR/TCID50 positive	Next collection qPCR/TCID50 results
101-3405-2001	Cycle 1 Day 1	Pre 1st dose	11600	1590	ND/NP	315000/ND
101-4402-2004	Cycle 2 Day 15	6h post 2nd dose	64200000	38900	ND/NP	4350000/3170
101-4402-2004	Cycle 2 Day 16	24hr Post 2^nd^ dose	4350000	3170	298/ND	BQL/ND
101-4901-2001	Cycle 1 Day 15	2wks Post 1st dose	3870000	4860	3060000/ND	219000/ND

ND, Not detected.

Of these 4 samples, one (101-3405-2001) was due to contamination as this was a positive detection prior to any RP1 dosing. In pt 101-4402-2004, two samples were positive for live RP1, but both these are collected within 24 hr of RP1 injection. The dressing samples at 24 hr was negative demonstrating that occlusive dressing acted as a barrier to prevent any dissemination of live RP1. The injection site sample from 4901–2001 was collected from a CSCC patient with large leaky lesion (**Supplementary Figure 3**). However, the dressing sample tested negative. No transmission to caregivers or close contacts was reported. Dressing samples were negative for live RP1, supporting the effectiveness as a barrier.

Some results (12.3%, 44/358) could not be determined mostly due to microbial contamination or non-availability of samples (**Table 5**; **Figure 4**). Among the live RP1-positive samples, only low levels of RP1 were detected (<10^5^ PFU).

During follow-up, RP1 DNA was detected in 14 of 272 samples. At 30 days after the last dose, 9.4% (12/127) of patients had RP1 DNA detected, while at 60 days only 1.8% (2/110) tested positive. All samples were negative for live RP1 by the 100-day follow-up. (**Table 5**; **Figure 4**).

### 3.6 RP1 detection on the exterior of injection site dressings

**Figure 5:**
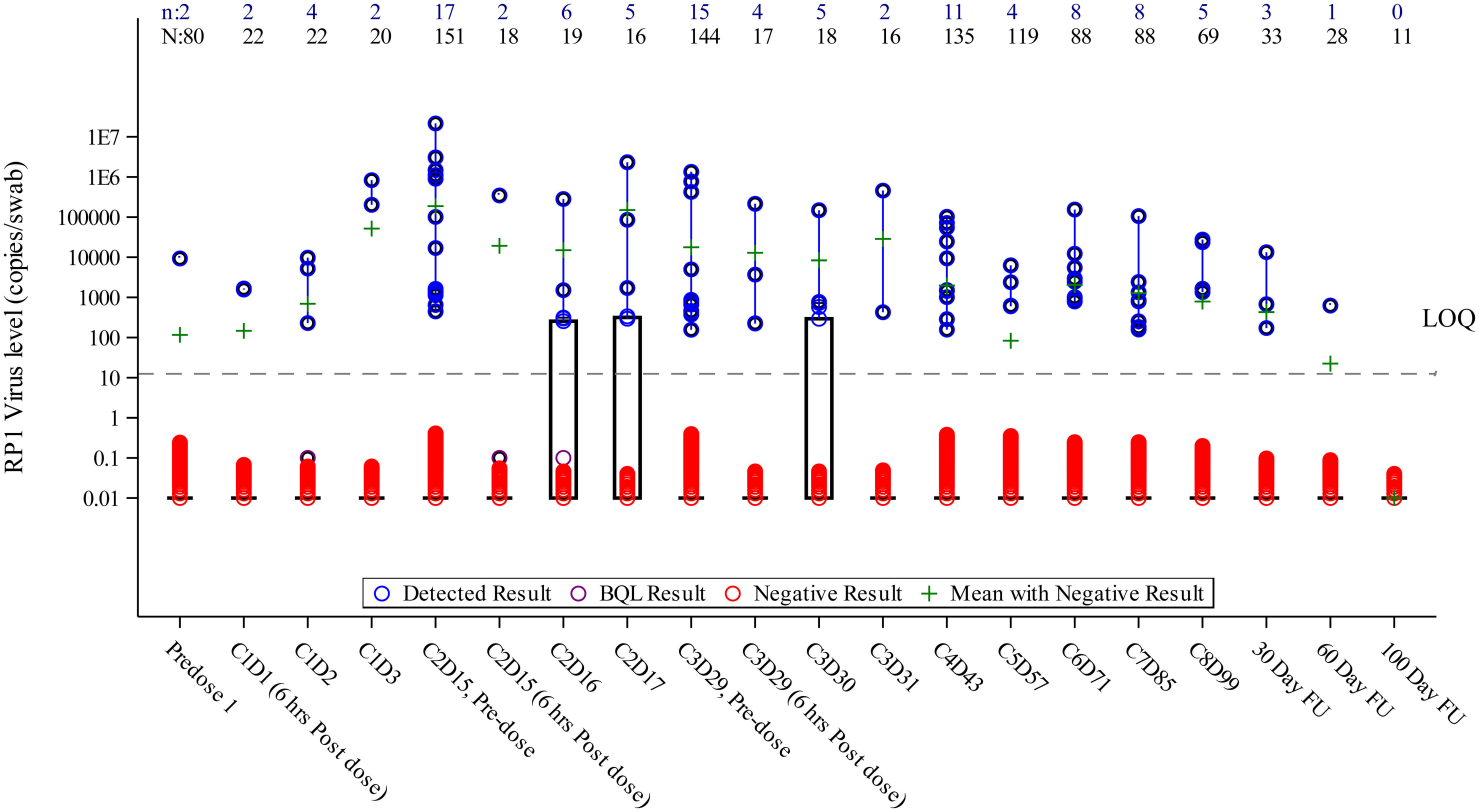
RP1 DNA detection on the exterior of injection site dressings. RP1 DNA detection in exterior of injection site dressings samples from all skin cancer cohorts across various study time points. “n” represents the number of patients with viral DNA levels equal to or above the limit of quantification (LOQ), while “N” indicates the total number of patients assessed at each time point. The study includes measurements taken at pre-dose, multiple post-dose time points, and follow-up visits, including Cycle 1 Day 1 (C1D1), Cycle 2 Day 15 (C2D15), Cycle 3 Day 29 (C3D29), Cycle 4 Day 43 (C4D43), Cycle 5 Day 57 (C5D57), Cycle 6 Day 71 (C6D71), Cycle 8 Day 99 (C8D99), 30 Day follow-Up (FU), 60 Day FU and 100 Day FU. Individual data points include detected results (blue circles), below quantification limit (BQL) results (purple circles), and negative results (red circles). Green plus signs represent the mean virus levels.

The incidence of RP1 DNA detection from the exterior of injection-site dressings was lower than that from injection-site samples, with only 43 of 207 (20.8%) patients and 106 of 1114 (9.5%) exterior dressing samples testing positive for RP1 DNA (**Table 7**; **Figure 5**; **Supplementary Figure 1**). Two samples tested positive for RP1 DNA at pre-dose cycle 1 day 1, likely due to cross-contamination, as no RP1 was administered before that visit. Lower incidence levels with low copy numbers (~10-fold) of RP1 DNA copies were generally detected on dressing samples compared to the injection site, demonstrating that the dressing was an effective barrier to prevent the dissemination of RP1 (**Table 7**; **Figures 4**, **5**).

**Table 7:** Patient and sample incidence of RP1 DNA detection on the exterior of injection site dressings.

	Patient incidence					Sample incidence			
	Baseline HSV-1 seronegative N=72 n1/n2 (%)	Baseline HSV-1 seropositive N=200 n1/n2 (%)	Baseline HSV-1 unknown N=6 n1/n2 (%)	Overall N=278 n1/n2 (%)		Baseline HSV-1 seronegative n3/n4 (%)	Baseline HSV-1 seropositive n3/n4 (%)	Baseline HSV-1 unknown n3/n4 (%)	Overall n3/n4 (%)
Dressing	14/58 (24.1)	28/145 (19.3)	1/4 (25.0)	43/207 (20.8)		31/341 (9.1)	74/754 (9.8)	1/19 (5.3)	106/1114 (9.5)

N, Number of patients in the analysis set; n1, number of patients with positive qPCR testing result; n2, number of patients with samples collected; n3, number of samples with positive qPCR testing result; n4, number of samples collected.

No significant differences were observed in the incidence of detectable RP1 DNA from the exterior of injection-site dressings between baseline HSV-1 seronegative and seropositive patients (**Table 7**; **Supplementary Figure 1**). Specifically, RP1 DNA was detected in 14 of 58 (24.1%) patients and 31 of 341 (9.1%) samples in baseline seronegative patients, compared to 28 of 145 (19.3%) patients and 74 of 754 (9.8%) samples in baseline seropositive patients.

During the follow-up visits, RP1 DNA was detected after the last dose in 3 of 33 (9.1%) samples at the 30-day follow-up visit and in 1 of 28 (3.6%) samples at the 60-day follow-up visit and none thereafter (**Figure 5**). RP1 DNA-positive exterior dressing samples (n) were further tested using the TCID50 assay for the presence of live RP1, and all were negative (**Table 7**; **Figure 5**).

### 3.7 RP1 DNA detection on oral mucosa/saliva

**Figure 6:**
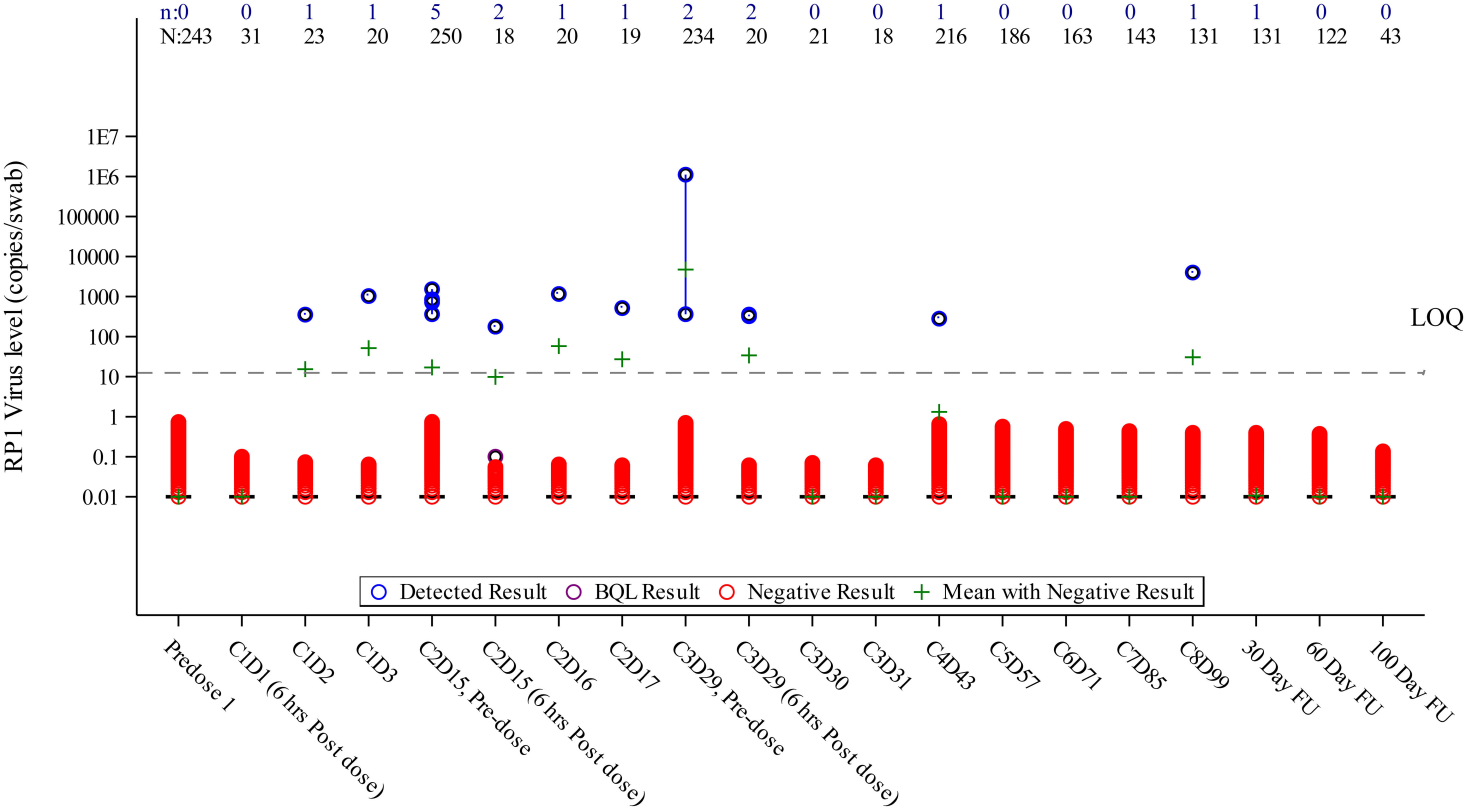
RP1 DNA detection in oral mucosa and saliva samples. RP1 DNA detection in oral mucosa/saliva samples from all skin cancer cohorts across various study time points. “n” represents the number of patients with viral DNA levels equal to or above the limit of quantification (LOQ), while “N” indicates the total number of patients assessed at each time point. The study includes measurements taken at pre-dose, multiple post-dose time points, and follow-up visits, including Cycle 1 Day 1 (C1D1), Cycle 2 Day 15 (C2D15), Cycle 3 Day 29 (C3D29), Cycle 4 Day 43 (C4D43), Cycle 5 Day 57 (C5D57), Cycle 6 Day 71 (C6D71), Cycle 8 Day 99 (C8D99), 30 Day follow-Up (FU), 60 Day FU and 100 Day FU. Individual data points include detected results (blue circles), below quantification limit (BQL) results (purple circles), and negative results (red circles). Green plus signs represent the mean virus levels.

Detection of RP1 DNA in oral mucosa/saliva swab samples was minimal, with only 16 of 272 (5.9%) patients and 18 of 2052 (0.9%) samples, and all being at low copy number (**Table 8**; **Figure 6**). Most of the RP1 DNA-positive oral mucosa/saliva swab samples were collected during the period of the first three doses of RP1. Further testing of those samples for live RP1 using the TCID50 assay showed that 8 of 18 (44.4%) were negative. The remaining 10 of 18 (55.6%) samples could not be conclusively analyzed due to microbial contamination in the culture likely due to the sample source on the mucosa (6 of 10), or due to sample non-availability (4 of 10).

**Table 8:** Patient and sample incidence of RP1 DNA detection in oral mucosa/saliva.

	Patient incidence				Sample incidence			
	Baseline HSV-1 seronegative N=72 n1/n2 (%)	Baseline HSV-1 seropositive N=200 n1/n2 (%)	Baseline HSV-1 unknown N=6 n1/n2 (%)	Overall N=278 n1/n2 (%)	Baseline HSV-1 seronegative n3/n4 (%)	Baseline HSV-1 seropositive n3/n4 (%)	Baseline HSV-1 unknown n3/n4 (%)	Overall n3/n4 (%)
Mucosa	2/69 (2.9)	14/197 (7.1)	0/6 (0.0)	16/272 (5.9)	2/563 (0.4)	16/1450 (1.1)	0/39 (0.0)	18/2052 (0.9)

N, Number of patients in the analysis set; n1, number of patients with positive qPCR testing result; n2, number of patients with samples collected; n3, number of samples with positive qPCR testing result; n4, number of samples collected.

RP1 DNA was detected at below quantitation levels (≤ 150 copies/swab) in 1 of 131 (0.8%) patients and 1 of 131 (0.8%) oral mucosa/saliva swab samples during the 30-day follow-up visit after the last dose. While TCID50 testing failed due to microbial contamination in mucosa samples it is unlikely that such low levels of RP1 DNA would yield detectable live RP1, as each PFU of virus typically corresponds to 50–100 copies of the RP1 genome. The mucosa sample collected at the subsequent 60-day follow-up visit tested negative for RP1 DNA, and it was suspected the previous positive result at 30 days post last dose was due to remnant RP1 DNA from a nearby injected lesion.

No differences were observed in the incidence of RP1 DNA detection in oral mucosa/saliva samples between HSV-1 seronegative and seropositive patients. The incidence of RP1 DNA detection in oral mucosa/saliva samples was 2 of 69 (2.9%) patients and 2 of 563 (0.4%) samples in baseline seronegative patients, compared to 14 of 197 (7.1%) patients and 16 of 1450 (1.1%) samples in baseline seropositive patients. RP1 DNA remained undetectable in all tested samples at the 60- and 100-day follow-up visits (**Table 8**; **Supplementary Figure 1**).

### 3.8 RP1 detection from areas of possible herpetic infection

Swab samples from 7 patients (8 samples in total) were collected due to possible herpetic infection. Two of these swab samples were taken from patient 1158-2011, who was HSV-1 seropositive at baseline, and patient 1122-2057, who was HSV-1 seronegative at baseline, both of whom experienced Grade 2 AESIs. The swab samples from both patients were collected due to presence of a suspected cold sore. Both samples tested negative for RP1 DNA confirming that these were not related to RP1. Only 1 of 8 swab samples tested positive for RP1 DNA. This positive sample was from patient 1152–2002 and was collected from a suspected herpetic skin lesion located in the right posterior auricular region. That same region (right posterior auricular) was injected with RP1–7 days earlier. Because of the proximity between the lesion and the injection site, it is likely that the positive RP1 DNA signal was due to contamination from the injection site. The TCID50 assay was negative. The patient subsequently withdrew from the study, and, therefore, no follow-up samples could be collected. All 7 other swab samples were negative for RP1 DNA.

### 3.9 Patient contacts exposure to RP1 and potential transmission

During each study visit, patients were asked about third-party exposure to RP1. Across all RP1 studies (IGNYTE, ARTACUS, and CERPASS) evaluated to date, 1211 responses have been received from 175 patients, of which 91 responses from 24 patients indicated that their close contact or caregiver came into direct contact with the RP1 injection site or dressings or cleaning swabs, however none reported symptoms of HSV-1 infection. These data demonstrate that the likelihood of RP1 transmission from patients to their close contacts is minimal.

## 4 Discussion

RP1 is engineered to selectively replicate in tumors but not in normal tissues (4, 14). Due to the modifications in the RP1 backbone the potential for RP1-related clinically-evident transmission to patients contacts (5) is therefore very low, as productive RP1 replication would not be expected to occur in normal tissues. Here we aimed to assess this by comprehensively studying the biodistribution, shedding, potential for transmission of RP1, and by investigation of any suspected cases of transmission in patients enrolled in the IGNYTE clinical trial.

RP1 DNA was detected at only low levels in blood, urine, and swab samples during treatment and was rapidly cleared after the first 48 hr. The timing of RP1 DNA detection in the blood aligned with the expected kinetics of RP1 replication in the tumor (within the first 1–3 days) (15, 16), with higher levels of RP1 DNA observed immediately after injection (up to 48 hours), followed by a reduction. Detection in urine was minimal (0.7% of patients and 0.2% of samples), demonstrating a negligible potential for environmental dissemination via urine.

The highest incidence of RP1 DNA detection was observed at the injection site (approximately 35% of swab samples up to 15 days post-injection), reflecting localized RP1 replication with live RP1 only detected immediately (up to 24 hours) after injection and only in one patient. RP1 DNA detection from the exterior of injection-site dressings was lower (9.5% of samples), demonstrating that the occlusive dressings acted as an effective barrier to RP1. Live RP1 was detected in only 1.1% of injection-site swabs and not in any other samples. Most often, only low copy numbers of RP1 DNA were detected. However, as the presence of DNA does not equate to the presence of live RP1, RP1 DNA positive samples were also tested by the TCID50 assay for the presence of live RP1. The majority of RP1 DNA positive samples were negative for live RP1, including all of the samples from the 7 patients (8 samples in total) who had the suspicion of possible herpetic infection.

The genetic modifications to RP1 prevent productive replication in normal tissues, which, along with its rapid clearance from blood and urine, likely contribute to its favorable safety profile (3). These findings are also consistent with previously reported data on other oncolytic immunotherapies, including talimogene laherparepvec (T-VEC) (17). T-VEC became the first FDA-approved oncolytic immunotherapy for cancer in 2015 and has now been in clinical use for over a decade. T-VEC is a genetically modified herpes simplex virus type 1 designed to selectively replicate in tumors and stimulate anti-tumor immunity, much like RP1. Across extensive clinical experience, T-VEC has demonstrated an excellent record in terms of infectious safety, with limited shedding, no significant transmission events, and only mild, localized side effects at the injection site (17, 18).​ The similar design features of T-VEC and RP1, both involving genetic modifications that restrict replication to tumors, support the expectation of a favorable infectious safety profile for RP1. This direct comparison with T-VEC is helpful, as its long-term record with the FDA affirms that appropriately engineered oncolytic viruses can be deployed safely, with minimal risk to both patients and contacts. Like T-VEC, RP1 also has an intact HSV-1 thymidine kinase gene and thus retains sensitivity to acyclovir. There were no systemic HSV-1 infections in patients or any reports of confirmed HSV-1 infections in patient’s contacts or care givers. Further, the overall incidence of suspected HSV-1 infections in the current study, including with wild type HSV, were minimal, probably due to the development of HSV-1 IgG antibodies in seronegative patients and increase in HSV-1 IgG in seropositive patients. This may have contributed to protection against subsequent HSV-1 infections and might have provided a vaccination effect against HSV-1. The shedding profile of a live biological agent should be taken into consideration prior to assigning Biosafety Level (BSL) classification and implementing associated biosafety requirements at clinical sites. Shedding characteristics including the duration, routes, magnitude, and infectivity of shed material influence the potential for risk to healthcare workers, other contacts, and the environment. BSL classification and related clinical site regulations should therefore be informed by a comprehensive assessment of shedding and related data to ensure that risk mitigation measures are proportionate and evidence-based, while remaining operationally feasible. The data reported here for RP1, indicates that as for T-VEC, standard infection control procedures as are routinely employed in clinical practice are sufficient to mitigate for any low potential risk which may be perceived for the clinical implementation and use of RP1.

Limitations of this study include that extensive long-term follow-up to assess persistence of RP1 genome in patients has not yet been performed beyond the study duration, and the potential for microbial contamination of samples affecting some of the analyses. Additionally, because the study was conducted in a controlled clinical environment, it remains possible that the low potential for shedding and transmission observed may not be reflected in broader real-world use. An follow-up study is ongoing intended to provide further data that will help address this.

## 5 Conclusion

This evaluation of the biodistribution, shedding, and potential for transmission of RP1 supports previous conclusions from preclinical studies, and the IGNYTE trial with RP1, that there is minimal potential for the transmission of RP1 to patient contacts.

## Data Availability

The original contributions presented in the study are included in the article/**Supplementary Material**. Further inquiries can be directed to the corresponding author. Replimune Inc, Woburn, MA was involved in the study design, data collection, interpretation of data, writing of this article, and the decision to submit for publication. Sample testing was performed by Viracor Eurofins, Lenexa, KS. and data analysis was conducted by eClinical, Mansfield, MA.

## Supplementary material

Supplementary Figure 1 incidence of viral DNA in seropositive vs seronegative patients. **(A)** Blood, **(B)** Dressing, **(C)** Mucosa, **(D)** Injection site, **(E)** Urine.

Supplementary Figure 2 Average RP1 virus copies/mL in blood in seronegative and seropositive patients, C=cycle of treatment, D=day since first cycle first dose. N, number of patients with samples collected at each visit timepoint; n, number of patients with RP1 DNA levels equal to or above the limit of quantification (LOQ); Multiple unpaired t-tests for each timepoint comparison between seronegative and seropositive patients do not reveal any significant differences.

Supplementary Figure 3 Example of a patient with a leaky lesion where the injection site swab sample tested positive for the presence of live virus enrolled in anti-PD-1 naïve non-melanoma skin cancer tumor at screening and 9 weeks of VO treatment.
